# Release of PACAP-38 in episodic cluster headache patients – an exploratory study

**DOI:** 10.1186/s10194-016-0660-7

**Published:** 2016-07-30

**Authors:** Bernadett Tuka, Nikoletta Szabó, Eszter Tóth, Zsigmond Tamás Kincses, Árpád Párdutz, Délia Szok, Tamás Körtési, Teréz Bagoly, Zsuzsanna Helyes, Lars Edvinsson, László Vécsei, János Tajti

**Affiliations:** 1 Department of Neurology, Faculty of Medicine, University of Szeged, Semmelweis u. 6, Szeged, H-6725 Hungary; 2 MTA-SZTE Neuroscience Research Group, Semmelweis u. 6, Szeged, H-6725 Hungary; 3 Department of Pharmacology and Pharmacotherapy, Faculty of Medicine, University of Pécs, Szigeti u. 12, Pécs, H-7624 Hungary; 4 János Szentágothai Research Center, University of Pécs, Ifjúság útja 20, Pécs, H-7624 Hungary; 5 MTA-PTE NAP B Pain Research Group, University of Pécs, Szigeti u. 12, Pécs, H-7624 Hungary; 6 Department of Clinical Sciences, Division of Experimental Vascular Research, Lund University, Sölvegatan 17, BMC A13, Lund, 22184 Sweden

**Keywords:** Episodic cluster headache, Plasma PACAP-38-LI, Inter-bout period, Ictal phase

## Abstract

**Background:**

Activation of the trigeminal-autonomic reflex, involving the trigeminal ganglion, the superior salivatory nucleus and the sphenopalatine ganglion (SPG) is crucial in the pathophysiology of cluster headache (CH). Since pituitary adenylate cyclase-activating polypeptide-38 (PACAP-38) is present both in the SPG and the trigeminal ganglion (TG) and its role in migraine has been described, our aim was to determine the plasma PACAP-38 levels in different phases of episodic CH (ECH).

Peripheral cubital fossa blood samples were taken during the ictal and inter-bout periods of male ECH patients and from age-matched healthy controls (*n* = 9). Plasma PACAP-38-like immunoreactivity (LI) was measured with specific and sensitive radioimmunoassay.

**Findings:**

Significantly lower plasma PACAP-38-LI was detected in the inter-bout period of ECH patients than in healthy controls. However, PACAP-38 was significantly elevated in the plasma during CH attacks as compared to the inter-bout phase in the same subjects (*n* = 5).

**Conclusions:**

This exploratory study suggests that PACAP-38 may be released during the attacks of ECH. Further patients and long-term follow-up are necessary to reveal its function.

## Background

Cluster headache (CH) is a primary, trigeminal-autonomic headache disorder, manifesting in periodically occurring unilateral (supra/orbital, temporal) severe pain attacks in association with intense ipsilateral autonomic symptoms. The trigeminal-autonomic reflex (TAR) and the hypothalamic system (HS) are thought to play important roles in the mechanism of CH [1]. The significance of TAR is based on the structural and functional connections between the trigeminal ganglion (TG), the superior salivatory nucleus being the parasympathetic nucleus of the facial nerve, the sphenopalatine ganglion (SPG) and otic ganglion (OG). CH attacks involve activation of the TG and the SPG resulting in release of neuroactive neuropeptides such as calcitonin gene-related peptide (CGRP) and vasoactive intestinal polypeptide (VIP) [2]. These molecules reach the cranial vessels, the lacrimal glands and the nasal mucosa via the postganglionic fibers, leading to conjunctival injection, eyelid and nasal edema, and rhinorrhea. The circadian and circannual periodicity of the attacks is attributed to the activation of the HS.

Neurochemical studies have revealed that the VIP-related neuropeptide pituitary adenylate cyclase-activating polypeptide-38 (PACAP-38) co-localize [3] in the SPG and the OG [4], however some PACAP-38-immunoreactivity was also seen in the TG [5, 6]. It has recently been described that PACAP-38 mediates the activation of the trigeminovascular system (TS), and exerts modulatory function in the sensitization process and migraine headache [7–10]. Human studies have confirmed that intravenously administered PACAP-38 induces migraine-like headache with marked vascular effects in migraine patients without aura [11, 12], and that PACAP-38 concentrations in the systemic circulation are significantly altered depending on the phases of migraine headache [13, 14].

Therefore, PACAP-38 is likely to be an important factor in migraine pathophysiology; however, there is less evidence concerning its function in other primary headache disorders such as CH. It is suggested that PACAP-38 may be involved in the evolution of trigeminal-autonomic cephalalgias, since this peptide was presented in the TS [8, 9, 15, 16], and also in the SPG and OG [17, 18] of humans and rats. Moreover there is a clear increase of both trigeminal (CGRP) and parasympathetic (VIP) neuropeptides during CH attacks which is normalized when the pain was controlled in CH [19, 20], and it appears that activation of the VPAC1 and PAC1 receptors influences the TAR, the parasympathetic cranial outflow [17, 21]. Based on these data, our aim was to determine if CH patients are associated with alterations in plasma PACAP-38-like immunoreactivity (LI) in the inter-bout period and during the attack in patients suffering from ECH in comparison with age- and gender-matched healthy volunteers.

## Materials and methods

### Participants

Nine male ECH patients treated in our outpatient clinic (without other known neurological diseases) were selected in accordance with the criteria of the Headache Classification Committee of the International Headache Society. Nine age-matched male healthy volunteers screened for non-reported/non-treated headaches served as controls (age of healthy control subjects: 40.1 ± 12.0 years old, age of EHC patients: 40.3 ± 9.6 years old). Coexisting depression in patients was ruled out by Hamilton’s depression scale. A detailed questionnaire was used to assess the features of the headache disorder. The clinical and demographic data are summarized in Table 1.

**Table 1:** Demographic data and clinical features of CH patients

Questionnaire	Mean values and features of ECH patients (*n* = 9)
Body mass index:	28.5 ± 5.6
Duration of cluster headache disease:	7.0 ± 3.8 years
Frequency of cluster episodes and duration of cluster episode:	2.9 ± 0.8 attack episodes/year
	5.0 ± 3.1 weeks
Sessions and seasonality of headache episodes:	in the evenings/at nights (*n* = 4), at dawns (*n* = 3) and daytime (*n* = 2)
	in spring and summer (*n* = 4), in changing seasons (*n* = 4), in winter (*n* = 1)
Intensity and characteristic of pain:	very strong (*n* = 6) and severe (*n* = 3)
	splitting/throbbing (*n* = 5), sharp/stabbing (*n* = 4) headache
Localization and side of pain:	orbital, supra/periorbital, temporal
	right (*n* = 6) and left (*n* = 3) side
Accompanying symptoms:	conjunctival injection, lacrimation, ptosis and eyelid edema, nasal congestion, rhinorrhea, vasodilation, facial redness and flushing
Lifestyle habits:	non (*n* = 4), medium (*n* = 2) and excessive (*n* = 3) smokers, moderate caffeine consumers (*n* = 9)
Most commonly used treatments of ECH patients in the bout periods:	O_2_ therapy, sumatriptan, nonsteroidal anti-inflammatory drugs (e.g. indomethacin and diclophenac)
Previous attack episode before the inter-bout blood sampling:	2.8 ± 1.0 months
Beginning of the attack episode during the ictal blood sampling:	6.4 ± 4.6 days
Duration of headache attack until the ictal sampling:	2.3 ± 0.6 h
Headache attack frequency per day in a bout and duration of headache attack:	1.2 ± 0.7 headache attack/day in a bout
	2.3 ± 0.7 h

### Study design sampling procedures

The study was approved by the Ethics Committee of the Faculty of Medicine, University of Szeged (87/2009). All participants gave their written informed consent in accordance with the Declaration of Helsinki. There were no restrictions regarding food and drink intake. The study was performed between 2010 and 2012 years. Blood samples (6 ml per subject) were taken from the cubital vein in the routinely used clinical blood sampling plastic tubes containing 12 mg ethylenediaminetetraacetic acid (BD Vacutainer, Becton Dickinson Hungary Plc., Hungary) and 1200 IU peptidase inhibitor, aprotinin (Gordox, Richter Gedeon Plc., Hungary). A single blood sample was taken in supine position after a rest of 10 min from the controls, whereas samples were taken from ECH patients during the acute spontaneous attack (when they produced typical symptoms of CH [22]) and/or in the absolute attack-free, inter-bout period (out of the cluster episode—by more than a month) between 8 a.m. and 4 p.m. Samples from both periods (during attack and outside attack) could be obtained in 5 cases (it was unintentional selection), which could be used in self-controlled comparison. Patients were asked not to start their usual treatment before sampling even during the ictal sampling. Exact date and time of blood samplings are presented in the Table 2.

**Table 2:** Detailed data of blood samplings in ECH patients: date and time, concentrations of PACAP-38 and therapyᅟ

*Patients*	*Date and time of inter-bout sampling*	*Inter-bout PACAP-38 plasma level (fmol/ml)*	*Last previous attacks (months ago)*	*Date and time of ictal sampling*	*Ictal PACAP-38 plasma level (fmol/ml)*	*Beginning of bout period (days) and attacks (hours) before the ictal sampling*	*Applied therapy*
*ECH-P1*	*06 June 2010 03:30 pm*	22,70	3.0	*15 May 2011 07:35 pm*	31,00	*Since 14 days, 3 h*	*O*_*2,*_ *Sumariptan*
*ECH-P3*	*13 June 2010 01:15 pm*	28,80	2.5	*27 June 2010 07:55 pm*	28,80	*Since 2 days, 1.5 h*	*O*_*2*_
*ECH-P4*	*11 July 2010 01:15 pm*	20,91	5.0	*16 Aug 2010 08:00 am*	26,00	*Since 7 days, 2.5 h*	*O*_*2*_ *, Sumatriptan*
*ECH-P8*	*10 Sept 2010 08:30 am*	24,40	2.0	*15 Oct 2010 02:45 pm*	27,30	*Since 5 days, 2.5 h*	*O*_*2*_
*ECH-P9*	*19 Sept 2010 10:30 am*	25,20	2.5				*NSAIDs*
*ECH-P10*	*28 Nov 2010 01:00 pm*	24,50	1.5				*NSAIDs*
*ECH-P11*	*12 Jan 2011 10:00 am*	22,20	3.0				*NSAIDs*
*ECH-P12*	*29 Jan 2012 12:30 pm*	23,37	3.5	*11 Oct 2011 01:20 pm*	29,1	*Since 4 days, 2.0 h*	*O*_*2*_ *, Sumatriptan*
*ECH-P13*	*19 Dec 2011 09:00 am*	25,06	2.5				*O*_*2*_ *, Sumatriptan, NSAIDs*
*Mean ± SD*		24.1 ± 2.3	2.8 ± 1.0		28.4 ± 1.9	6.4 ± 4.6 days 2.3 ± 0.6 h	

### Analysis of PACAP-38 in plasma and data analysis

Blood collection, centrifugation, storage and PACAP-38 measurement were carried out with specific and sensitive radioimmunoassay (RIA). Briefly, following centrifugation of the plasma samples (2000 rpm at +4 °C for 10 min), the peptide was extracted from the plasma into three volumes of absolute alcohol. After precipitation and a second centrifugation, the samples were dried under a nitrogen flow and resuspended in 300 μl of assay buffer before RIA determination. The assay was prepared in 1 mL 0.05 mol/L (pH 7.4) phosphate buffer containing 0.1 mol/L sodium chloride, 0.25 % (w/v) bovine serum albumin and 0.05 % (w/v) sodium azide. The tracer mono-^125^I-labeled PACAP24–38 (5000 cpm/tube, 124 Bq/1.72 fmol) was prepared in our laboratory. Ovine PACAP38 was used as a RIA standard ranging from 0 to 1000 fmol/mL. The PACAP-38 antiserum (88111-3, dilution 1:10000 obtained from Abcam UK), the RIA tracer and the standard or unknown samples were measured into polypropylene tubes with the assay buffer. After incubation for 48–72 h at +4 °C, the antibody-bound peptide was separated from the free peptide by the addition of separating solution. Following centrifugation (3000 rpm at +4 °C for 20 min), the contents of the tubes were gently decanted and the radioactivity of the precipitates was measured in a gamma counter (Gamma, type: NZ310). The PACAP-38 concentrations of the unknown samples were read from calibration curves.

### Statistical analysis

Data are expressed as median, interquartile range (IQR), minimum and maximum or individual values on the graphs. The distributions of data populations were checked with the Shapiro-Wilk normality test and the Levene’s test for analysis of the equality of variances was also applied.

Groups were compared with two sample unpaired and paired t-tests where appropriate via Monte-Carlo permutation (with 10000 random permutations) due to the small number of subjects, the unequal variances and the non-Gaussian distribution of the data of the control group. Correlations were revealed by the Pearson’s test. Statistical analyses were performed using the R software (R Development Core Team, 2002). Significance was accepted at *p* < 0.05.

## Findings

### Differences in plasma PACAP-38-LI between ECH patients and healthy controls

The level of plasma PACAP-38-LI in the inter-bout phase of ECH patients was significantly lower (*n* = 9; median = 24.4 fmol/ml, IQR = 2.68) than in age and gender matched healthy volunteers (*n* = 9; median = 30.5 fmol/ml, IQR = 8.84; *p* < 0.026).

**Fig. 1:**
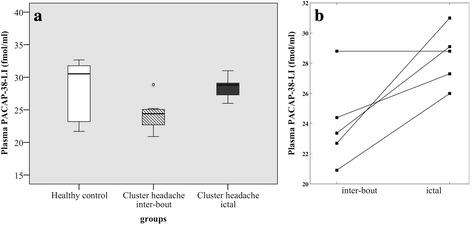
Plasma PACAP-38 concentrations in healthy control subjects and ECH patients (**a**), and changes of PACAP-38 levels in ECH patients between their inter-bout and ictal periods (**b**) (*n* = 5-9/group)

Plasma samples from patients during an attack in the cluster episode exhibited higher PACAP-38 concentrations (*n* = 5; median = 28.8 fmol/ml, IQR = 3.40) as compared to inter-bout samples (*n* = 9; median = 24.4 fmol/ml, IQR = 2.68), but these groups were not statistically compared because of the partly dependent data between the self-controlled, paired data (*n* = 5) and the other inter-bout data (*n* = 4). No difference was found when ictal samples (*n* = 5; median = 28.8 fmol/ml, IQR = 3.40) were compared to healthy controls (*n* = 9; median = 30.5 fmol/ml, IQR = 8.84; *p* < 0.965) (Fig. 1/a). When we compared only those subjects that delivered plasma samples collected from both periods, a significant elevation was detected in ictal compared to the corresponding inter-bout samples in a paired comparison as well (*n* = 5; median = 24.4 fmol/ml, IQR = 5.20; *p* < 0.032). As seen in the illustration four patients showed a clear increase from inter-bout to ictal sampling and only one patient was with unchanged value of PACAP-38 (Fig. 1/b).

### Positive correlation between inter-bout plasma PACAP-38 concentration and the age of patients

**Fig. 2:**
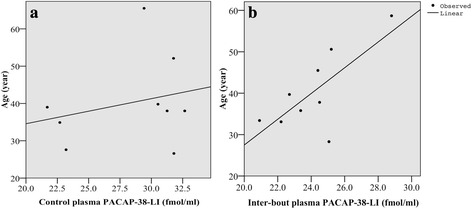
Association between the age of healthy control subjects and their plasma PACAP-38 level (**a**), and the age of the ECH patients and their inter-bout plasma PACAP-38 level (**b**) (*n* = 9/group)

A positive correlation was revealed between the age of ECH patients and the inter-bout plasma PACAP-38-LI (*n* = 9; *p* < 0.026, *r* = 0.728) (Fig. 2/b). No correlation was found between the age of healthy controls and their plasma PACAP-38-LI (*n* = 9; *p* < 0.520, *r* = 0.248) (Fig. 2/a). There were no correlations between PACAP-38-LI and the clinical parameters of ECH patients (data not shown).

## Discussion

This is the first study investigating alterations in plasma PACAP-38 concentration in CH patients; however limitation of our study is the sample size of ECH patients, especially the sample size of ictal blood samples. Our results agree with observations in plasma samples from migraine patients, obtained in the cubital fossa [11, 13] or the external jugular vein [14].

The source of PACAP-38 is unclear but two sites are to be discussed, the parasympathetic ganglia SPG and OT, and the TG, because neurons in ether contain PACAP-38. It is difficult to separate these sources of circulation PACAP-38, because they are intermingled. The stimulation of the superior sagittal sinus elicits release of both CGRP from the TG and VIP from the SPG/OT ganglion and the destruction of the trigeminal nerve aborts their release [10]. This shows interplay between the sensory and parasympathetic systems. It has been revealed that PACAP-38 may induce migraine-like attacks while VIP does not [11, 21, 23]. The role of PACAP-38 seems to differ from VIP, but there are similarities concerning theirs effects in the peripheral cutaneous nociception, which may be mediated by VPAC receptors [24].

Detailed study on distribution revealed that PACAP co-localize with CGRP in dura mater sensory fibers. The supply of parasympathetic innervation of the dura is scant as compared to that of the cerebral circulation [25]. Thus, it is possible that in both migraine and in CH the PACAP-38 release emanates mainly from the trigeminal system.

Our speculation is that the formation of PACAP-38 is depleted during the inter-bout period in certain nerve structures, which results in lower plasma PACAP-38 level outside the attacks of CH. Subsequently the chronically reduced peptide level may be a trigger for sensitization and altered nociception. During the evolution of nociception the concentration of PACAP-38 can increase and it exerts vasodilation, which is an essential trigger in conjunction with other unknown factors to the development of pain. During the attacks the level of PACAP-38 can reach the normal/control level, which may be a compensatory mechanism, but several further investigations are needed to affirm this theory.

It is also evidenced that the meningeal vasculature likely contributes to the propagation of the headache cascade of symptoms, but the vasodilator ability of PACAP-38 in these circumstances is questionable. It was showed that the PACAP-38 has high potency to induce dilation on pressurized rat meningeal arteries [26], while another study did not find any discernible effect of PACAP-38 on neither rat nor human samples [27].

Amin et al. have detected significantly elevated plasma PACAP-38 level following the PACAP-38 infusion in those migraine patients without aura, who later experienced migraine-like attack [11]. Although the half-life of this peptide in plasma is short (minutes), it was suggested that the administered PACAP-38 would induce long-lasting extracranial vasodilation and neurogenic inflammation and thereby provoke a migraine attack. The nature of such a mechanism is still unclear. An alternative mechanism might be that low levels of PACAP-38 is entering the CNS and interferes with central sensory mechanisms active in the migraine or CH brain.

Nevertheless, it is important to mention that elevation was detected in the plasma PACAP-38 level both in human migraine studies [11, 14] and experimentally, when the activation of the TS was provoked with electrical or chemical methods [7, 14]. However, these studies could not investigate the difference between the interictal and normal, healthy plasma PACAP-38 levels.

The main factor determining the levels of PACAP-38 in the systemic circulation is unidentified; however, it may originate from endogenous release, damaged elimination, or *de novo* synthesis [11]. Significant amount of peptide can be released from the peripheral (i.e., cranial meninges) and central branches (i.e., trigeminal caudal nucleus) of the TG and other PACAP-38-containing structures of the nervous system (i.e., SPG/OT) during the beginning of the headache. The activation of TAR is certainly involved in the neuropeptide overflow, which might result in middle meningeal artery vasodilation, mast cell degranulation, and peripheral and central sensitization; concomitantly it contributes to the development of headache [11, 13, 28].

Although PACAP-38 has not been previously investigated in CH, the concentrations of CGRP, VIP and substance P (SP) have already been examined. Regarding VIP a clinical study has revealed elevated VIP-like immunoreactivity in saliva of CH patients during attack period as compared with the VIP levels during the interictal phase [29]. Moreover, significantly increased CGRP and VIP levels were found in the plasma obtained from the ipsilateral external jugular vein during spontaneous attacks of ECH patients [19], and in the extracerebral circulation both during spontaneous and nitroglycerine-induced attack phase of CH [20, 30], whereas lower plasma SP levels were detected during histamine-induced and spontaneously occurring CH attacks when compared with controls [31]. Of note, similar alterations were found considering these neuropeptides in migraineurs, which parallels the concordant changes of PACAP-38-LI observed in migraine and CH disorders.

Additionally, it should be noted that the role of PACAP in CH may also be emphasized by the evidences, which showed that PACAP can modulate the melatonin synthesis, subsequently the circadian and circannual rhythm in animals (including mammals) [32–34]. However, we did not find any correlation between the plasma concentration of PACAP-38 and the date and time of blood sampling.

Moreover, we detected a positive linear correlation between the inter-bout plasma PACAP-38 level and the age of ECH patients, which suggests that older age is associated with smaller decrease in the concentration of PACAP-38 outside of cluster period. Our data can be related to clinical studies, which concluded that the development of chronic CH is much less frequent in older than in younger patients [35, 36], if we consider that a lower inter-bout plasma PACAP-38 concentration can be a triggering factor of attacks. However, it needs further cases and data collection to affirm our hypothesis regarding this association.

## Conclusion

Our pilot study provides the first evidence supporting that PACAP-38 may influence the course of CH. Similar changes of this peptide in migraine suggest that PACAP-38 might serve as a marker of primary headache conditions. Further investigations are necessary to determine the exact functions, targets and signaling pathways of PACAP-38.

## Abbreviations

CGRP, calcitonin gene-related peptide; CH, cluster headache; ECH, episodic cluster headache; EDTA, ethylenediaminetetraacetic acid; HS, hypothalamic system; LI, like immunoreactivity; OG, otic ganglion; PACAP-38, pituitary adenylate cyclase-activating polypeptide-38; RIA, radioimmunoassay; SP, substance P; SPG, sphenopalatine ganglion; TAR, trigeminal-autonomic reflex; TG, trigeminal ganglion; TS, trigeminovascular system; VIP, vasoactive intestinal polypeptide
